# Homœopathy in Spain

**Published:** 1883-08

**Authors:** El Palvarez

**Affiliations:** Madrid


### HOMŒOPATHY IN SPAIN.

DR. EL PALVAREZ, MADRID.

In Spain, Homœopathy is in a prosperous state, counting eight hundred homœopathic doctors that practice Hahnemannian therapeutics with entire freedom, unmolested by the government or by legal authorities—preparing and giving the medicaments themselves to the sick. Notwithstanding this, there are two large homœopathic pharmacies, one in Madrid and the other in Barcelona, where the medicines are to be found for the use of those homœopathic doctors who do not wish to prepare it themselves, and for the public benefit. The greatest number of homœopathists are found in the populations of Madrid and Barcelona.

The homœopathic doctors in Spain are divided into two parties, those using high and those using low dilutions—those using high dilutions being in the majority. They employ the high potencies of Jenichen, that his successor, Dr. Rentsch, of Wiemar, Germany, sends them, and these are the only ones known in Spain. Dr. Fincke would do well were he to give to the Spanish high-dilution party the knowledge of his potencies.

There is only one homœopathic society in Spain, "The Madrid Hahnemannian Society," founded in Madrid in 1845. It has published since 1846 a monthly periodical, that since 1860 has been entitled "*El Criterio Medico,*" said corporation supporting a free daily homœopathic dispensary for chronic diseases. It is largely attended, having seven or eight thousand patients annually. The Society holds two monthly sessions, having for its President Dr. Zoilo Perez, an old and illustrious homœopathic doctor, who is a diplomat at court and a member of the Spanish Parliament.

The Madrid Hahnemannian Society was founded by a public subscription. A homœopathic hospital, for acute diseases, excepting the contagious, was opened to the public in 1879, and has forty-eight beds. It is directed and administered by a body of managers and supported by a legacy left by Dr. Nuñez, by private donations, a public monthly subscription, and an annual subsidy of $4,800 from the Spanish Government. This subsidy is used to assist in defraying the expenses of the homœopathic school that has been established in said hospital, and which is authorized by the government.

The school is composed of four professorships, consisting of the following:

1st. Institutes and Homœopathic Medical Pathology.

2d. Pure Materia Medica.

3d. Homœopathic Medical Clinics, for men.

4th. Homœopathic Medical Clinics, for women.

Each chair is held by a professor, having an assistant professor as a substitute in case of illness or absence. Numerous alumni are annually matriculated, that after taking the courses mentioned above undergo an examination for a degree, receiving from the faculty the Diploma of Homœopathic Doctor, signed by the Director and Secretary of the Hospital. Before an alumnus can matriculate he needs and must have a diploma or degree of medical surgeon, given by some Spanish university, or have taken some lectures at the Medical School of the Madrid University.

The Hospital, or board of managers and doctors, publish monthly a bulletin giving the statistics of the sick that enter the establishment, and the notable cases treated at the clinics.

The homœopathic doctors are highly esteemed and appreciated by the Spanish Government, from which they frequently receive favors and distinctions, particularly from the monarchial government; they also hold public professional offices. The highest Spanish classes, and also the people, protect the homœopathic school. Were it not for this, the homœopathic hospital would never have been founded, nor would it have obtained assistance from the government.

Other homœopathic societies have been founded at various epochs, the only one now existing being the Madrid Hahnemannian Society.

The same is true of the homœopathic journals, the ones now edited are *El Critero Medico*, published by the Hahnemannian, and the *Bulletin*, published in the last two years by the managers and doctors of the Hospital.

